# Evaluation of the phytochemical composition and protective activities of methanolic extracts of *Centaurea borysthenica* and *Centaurea daghestanica* (Lipsky) Wagenitz on cardiomyocytes treated with doxorubicin

**DOI:** 10.1080/16546628.2017.1344077

**Published:** 2017-07-06

**Authors:** Agnieszka Korga, Aleksandra Józefczyk, Grażyna Zgórka, Mateusz Homa, Marta Ostrowska, Franciszek Burdan, Jarosław Dudka

**Affiliations:** ^a^ Independent Medical Biology Unit, Medical University of Lublin, Lublin, Poland; ^b^ Department of Pharmacognosy with Medicinal Plant Unit, Medical University of Lublin, Lublin, Poland; ^c^ Department of Toxicology, Medical University of Lublin, Lublin, Poland; ^d^ Department of Human Anatomy, Medical University of Lublin, Lublin, Poland

**Keywords:** Centaurea borysthenica Gruner, *Centaurea daghestanica* (Lipsky) Wagenitz, flavonoids, phenolic acids, HPLC/PDA analysis, doxorubicin cardiotoxicity, oxidative stress

## Abstract

*Centaurea* L. is a genus of the family *Asteraceae* that comprises over 600 taxa. Representatives of the *Centaurea* genus were used as natural medications for many diseases. Methanolic-aqueous extracts from aerial parts of two *Centaurea* species: *C. borysthenica* Gruner and *C. daghestanica* (Lipsky) Wagenitz were studied for their polyphenolic composition and potential protective effect on cardiomyocytes treated with doxorubicin. Effectiveness of doxorubicin in cancer therapy is limited by a dose-dependent cardiotoxicity. Oxidative stress is a widely recognized mechanism of this phenomenon. One of the most important strategies has been an application of drug together with antioxidant agents. A cardioprotective effect of selected extracts of *Centaurea* species was suspected in this study. Cell viability, oxidative stress, and mitochondrial membrane potential analyses showed protective activity of the methanolic extract of *C. borysthenica* and *C*. *daghestanica* on rat cardiomyocytes treated with doxorubicin. Although *C. borysthenica* is more effective as a cardiomyocyte protective agent, in higher concentrations it weakened the drug activity. *C. daghestanica* extract did not change the doxorubicin efficacy in the evaluated experiment. Interestingly, both tested extracts were cytotoxic for myeloma cells. The detected antioxidant activity of the studied extracts can be used in the prevention of doxorubicin-induced cardiotoxicity.

## Introduction

*Centaurea* L. (common name: knapweed) is a botanical genus belonging to the *Asteraceae* family that contains over 600 species. These taxa are thistle-like flowering plants that are found only on north of the Equator, mostly in the Eastern Hemisphere (the Middle East and surrounding regions are particularly rich in these herbaceous species). Also, the Mediterranean Sea area exhibits a large diversity and abundance (over 200) of knapweeds, especially in Turkey, where this genus is represented by more than 180 species, including 120 that are native [1,2].

In Turkish folk medicine, the plants of *Centaurea* genus were used as natural medications for pain reduction, inflammation in rheumatoid arthritis, hyperthermia, headaches, and haemorrhoids. According to numerous reports, these taxa are a potential source of natural antioxidants used in prevention and treatment of diseases in which reactive oxygen species are involved [3].

Doxorubicin (DOX) has been used in anti-tumour therapy for almost 50 years. Apart from its efficiency, it also exhibits toxicity towards myocardium. DOX effectiveness in cancer therapy is limited by a dose-dependent, irreversible and progressive cardiotoxicity. Oxidative stress is a widely recognized mechanism of DOX-induced cardiotoxicity. The selective cytotoxic effect of DOX on the heart is associated among others with poor antioxidant defence of cardiomyocytes [4]. Oxidative stress has been shown to cause depolarization of the mitochondrial membrane, resulting in apoptosis [5,6]. ROS generation in heart is also the cause of necrosis, heart remodelling, and changes in cells metabolism which are observed in the presence of DOX [7–11]. One of the crucial strategies resulting in oxidative stress reduction has been an application of the combination of drug together with antioxidant agents [12].

However, recently, numerous phytochemicals have attracted the attention of the scientific world as regards to their cardioprotective activities [13]. For a few years, chemotaxonomic studies concerning the chemical composition of polyphenolic compounds in some representatives of *Centaurea* L. genus have been performed in the Department of Pharmacognosy with Medicinal Plant Unit (Medical University of Lublin), together with the investigation of their biological potential. On the basis of preliminary studies, the pharmacological effects of two *Centaurea* extracts obtained from *C. borysthenica* Gruner and *C. daghestanica* (Lipsky) Wagenitz were examined in this study. Methanolic-aqueous (3:7, v/v) extracts possessing the richest set of various polyphenolic ingredients have been used.

## Material and methods

### Chemicals and reagents

The majority of reference polyphenolic phytochemicals used for the HPLC analysis (apigenin, luteolin, hesperidin, taxifolin, scopoletin, chlorogenic acid, neochlorogenic acid, protocatechuic acid) were purchased from Sigma (St. Louis, MO, USA). Apigenin 7-*O*-glucoside, centaurein, eryodictiol, and cynaroside were obtained from ChromaDex (Santa Ana, CA, USA).

### Plant material

The plant material (blooming herbs of *C. borysthenica* (CB) and *C. daghestanica* (CD)) was collected in July and September 2012, in the Botanical Garden of the Department of Pharmacognosy with Medicinal Plant Unit, Faculty of Pharmacy, Medical University of Lublin (Poland). After collection, plants were immediately dried in an oven at a temperature not exceeding 35°C and powdered in the laboratory mill to 1 mm particles. The certified seeds of two *Centaurea* species were obtained from CD-Kärntner Botanic Centre of the Botanical Garden in Klagenfurt (Germany) and CB-Institute of Ecology and Botany (The Hungarian Academy of Sciences) of the Botanical Garden in Vácrátót (Hungary).

### Accelerated solvent extraction

The methanolic-aqueous extracts of two *Centaurea* species were examined. For obtaining the plant preparations, the accelerated solvent extraction (ASE) method was employed. Pulverized herb samples (1.0 g) were placed in the stainless-steel cells (10 mL) of the ASE 100 apparatus (Dionex, Sunnyvale, CA, USA) and the extraction process was performed using methanol–water (3:7, v/v) mixture of solvents. The following optimized extraction parameters were used: a temperature of 70°C, flush volume 60%, number of cycles 3, and a constant pressure of extraction equal to 100 bar. The extracts obtained were concentrated under reduced pressure and taken up in 10 mL of 30% (v/v) methanol in calibrated vials and subjected to further chromatographic and biological investigations.

### Solid-phase extraction and HPLC analysis

Before HPLC analysis, the extracts obtained were purified from ballast compounds (chlorophyll) using solid-phase extraction on Octadecyl JT Baker (Phillipsburg, NY, USA) 500 mg columns. Extract samples (3 mL) were passed onto the sorbent beds and washed with 9 mL of 30% (v/v) methanol. Combined eluates were collected in 20 mL calibrated flasks and after filtration (0.45 μm) they were chromatographically analysed for composition and the content of polyphenolic compounds (phenolic acids, flavonoids, and simple coumarins). The Agilent Technologies Model 1100 liquid chromatograph (Waldbronn, Germany) equipped with a PDA detector, autosampler, and an online vacuum degasser was used. The separation of compounds examined was done on a thermostated Zorbax Eclipse XDB C8 column (150 × 4.6 mm I.D., dp = 5 μm), working at 25°C, with gradient elution: A – water with 1% (v/v) acetic acid; B – acetonitrile (0 min, 10% B; 0–10 min, 10–14% B; 10–15 min, 14–22% B; 15–25 min, 22–30% B; 25–35 min, 30–35% B; 35–45 min, 35–55% B; 45–50 min, 55–60% B).

The identification of the compounds was performed by comparing retention times with those for reference substances using three analytical wavelengths (λ = 254, 280, and 325 nm). Calibration plots were obtained using six different concentrations (0.01, 0.025, 0.05, 0.075, 0.1, and 0.15 mg per 1 mL) of all standard methanolic solutions. A statistical analysis was performed based on the results of 3 independent assays (*n* = 3) determining the content of all polyphenolic constituents in the extract samples examined.

### Cell culturing

The culture of embryonic rat cardiomyocytes H9C2 and suspension of human peripheral blood myeloma CCL-155 (ATCC, USA) were performed in Dulbecco’s Modified Eagel’s Medium and RPMI-1640 Medium respectively (PAA, Austria) supplemented with 10% foetal bovine serum. Cells were incubated at 37°C with 5% CO_2_ in air atmosphere. Cells were incubated for 24 h with extract of CB and CD at concentrations of 1 mg/mL, 0.5 mg/mL, 0.1 mg/mL, and 0.05 mg/mL and the DOX at concentration of 5 µM. Controls were media with and without DOX. For investigation of solvent influence, cells were treated with methanol in 0.3% concentration in culture medium, which was the highest concentration used for cell treatment with plant extracts.

### Cytotoxicity analysis

DOX cytotoxicity was evaluated with the MTT test, using the MTT Cell Proliferation Assay Kit (Invitrogen, US). The test principle is based on live cells’ ability to reduce orange tetrazolium salt to water-insoluble purple formazan crystals. MTT solution (4 mg/mL) was added to the culture 24 h after DOX. Following 4 h of incubation, the medium with MTT was removed, and the crystals formed were dissolved in DMSO. The solution absorbance was measured at 540 nm, using PowerWave™ microplate spectrophotometer (Bio-Tek Instruments, USA). The measurements were performed in triplicate, and each cytotoxicity experiment was repeated five times.

### Mitochondrial membrane potential

Cells from each culture were re-suspended in 1 mL of warm buffered saline (PBS). Then the JC-1 dye in a concentration of 2.5 g/mL was added and the samples were incubated for 10 min at 37°C. The stained cells were centrifuged at 400*g* for 5 min, washed with warm PBS, re-suspended in a solution of DAPI (1 µg/mL), and then analysed in the NucleoCounter NC-3000 (Chemometec, Denmark). In normal cells, the negative charge is determined by the intact mitochondrial membrane potential, facilitates the accumulation of JC-1 in the mitochondrial matrix. At higher concentrations JC-1 forms aggregates and has red fluorescence. In cells with a reduced mitochondrial potential, JC-1 is localized in the cytosol in a monomeric fluorescent green form.

### Oxidative stress

Oxidative stress was evaluated under a fluorescence microscope, using Mito-Tracker Green FM and RedoxSensor Red CC-1 (Molecular Probes, USA). Mito-Tracker Green FM passively diffuses through the cell membrane and accumulates in active mitochondria, resulting in green fluorescence. The location of Redox Sensor Red CC-1 marker depends on the cytosolic redox potential of the cell – the marker can be oxidized in cytoplasm and accumulates in mitochondria, resulting in red fluorescence, or is transported to lysosomes and there it is oxidized, emitting a fluorescence signal. Cells were incubated with staining agents at 37°C for 10 min. After incubation, the cells were rinsed twice with the PBS solution and observed under the Nikon Eclipse Ti microscope. Stain colocalization in mitochondria was evaluated on photographs taken in two colour channels and superimposed using the NIS-Elements Imaging Software.

### Statistical analysis

The obtained data were statistically analysed using the STATISTICA 8.0 software (StatSoft, Poland). The results are expressed as mean ± standard deviation (M ± SD). The statistical significance of differences between control and the other groups was evaluated by Student’s t-test. The value of *p* ≤ 0.05 was considered as statistically significant.

## Results

### HPLC qualitative and quantitative analysis

**Figure 1.**
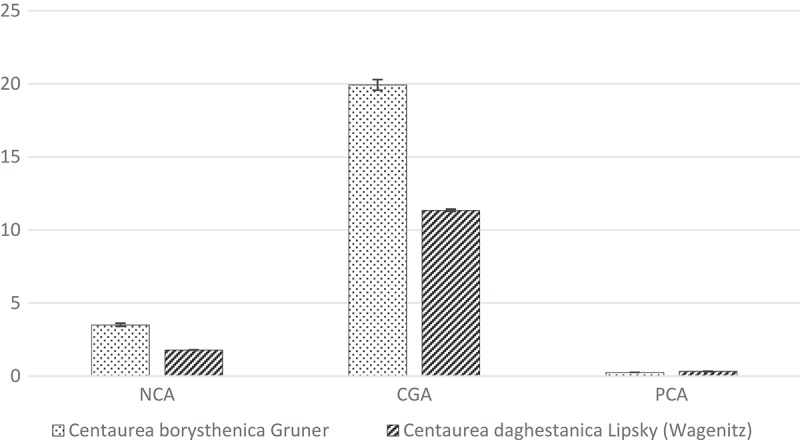
Phenolic acids content (mg/g dry substances) in tested extracts. NCA – neochlorogenic acid, CGA – chlorogenic acid, PCA – protocatechuic acid.

**Figure 2.**
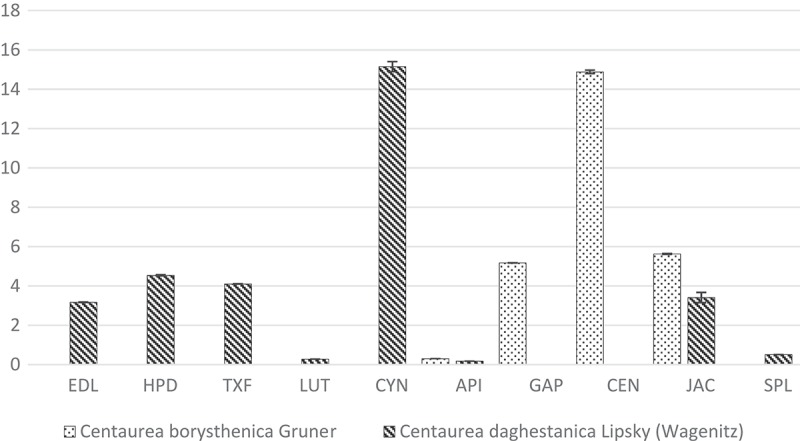
Flavonoids and scopoletin content (mg/g dry substances) in tested extracts. EDL -eryodictiol, HPD – hesperidin, TXF – taxifolin, LUT – luteolin, CYN – cynaroside, API – apigenin, GAP – apigenin7-O-glucoside, CEN – centaurein, JAC – jacein, SPL – scopoletin (coumarin).

In the group of phenolic acids three compounds were identified in both *Centaurea* species: the chlorogenic, neochlorogenic and protocatechuic acid. The chlorogenic acid was a predominant component in aerial parts of the investigated plant extracts. In CB, its amount was approximately 20 mg/g of dry weight and it was twice higher than in CD. The same proportions were obtained for the neochlorogenic acid. Its concentration was ~4.0 and ~2.0 mg/g, in CB and CD, respectively. The lowest amounts were documented for the protocatechuic acid (approximately 0.3 mg/g, for both CB and CD) (Figure 1). As regards the flavonoid compounds, cynaroside and luteolin (flavones), eryodictiol and hesperidin (flavanones) and taxifolin (flavanonol) were present only in CD, whereas centaurein and apigenin 7-*O*-glucoside (flavonols) were identified only in CB (Figure 2). Two flavonoids were reported in both species: apigenin (flavone) and jacein (flavonol). It was established that the predominant flavonoid constituents in both *Centaurea* species were cynaroside and centaurein, which reached the concentration level of 15 mg/g (dry weight). The simple coumarin – scopoletin – was identified only in CD, however its mean concentration was low (0.51 mg/g of dry weight) (Table 1).

**Table 1.** The content (mg/g, dry wt; SD; RSD) of phenolic acids, flavonoids and coumarins in extracts examined.

	Symbols of Species					
	CB			CD		
Compounds		SD	RSD		SD	RSD
CGA	19.92	0.04	0.37	11.35	0.07	0.65
NCA	3.51	0.1	0.32	1.79	0.01	0.5
PCA	0.26	0	1.11	0.32	0.01	0.27
EDL	-			3.17	0.01	0.22
HPD	-			4.54	0.03	0.75
TXF	-			4.1	0.01	0.33
LUT	-			0.28	0.02	0.58
CYN	-			15.15	0.26	1.7
API	0.3	0.03	1.10	0.18	0.03	1.72
GAP	5.17	0	0.06	-		
CEN	14.88	0.09	0.61	-		
JAC	5.63	0.02	0.27	3.41	0.26	0.77
SPL	-			0.51	0.01	1.35

API - apigenin; CEN - centaurein; CGA - chlorogenic acid; CYN - cynaroside; EDL - eryodictiol; GAP- apigenin-7-*O*-glucoside; HPD - hesperidin; JAC - jacein; LUT - luteolin; NCA - neochlorogenic acid; PCA - protocatechuic acid; SPL – scopoletin; TXF - taxifolin.

### Cardiotoxicity in vitro analyses

**Figure 3.**
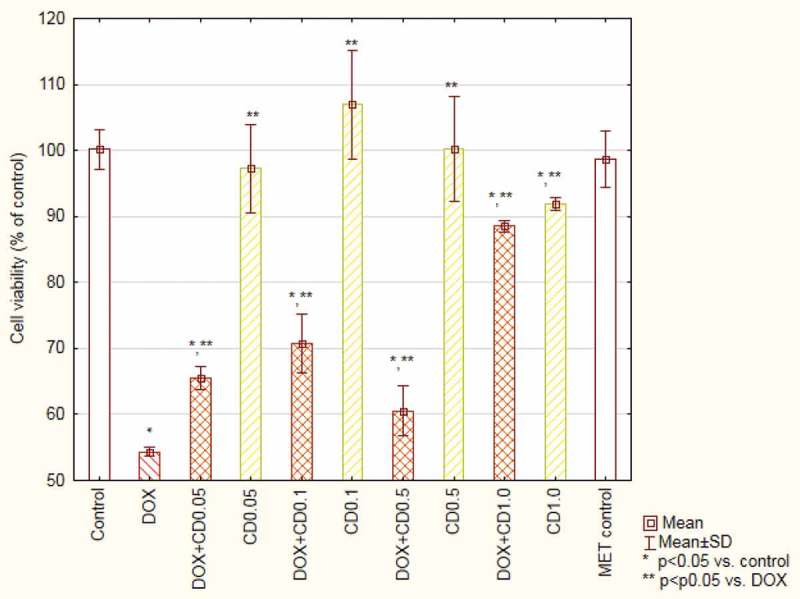
Viability of cardiomyocytes (based on MTT test results) treated with doxorubicin and *Centaurea borysthenica* Gruner extracts. DOX – doxorubicin, CB – *Centaurea borysthenica* Gruner.

**Figure 4.**
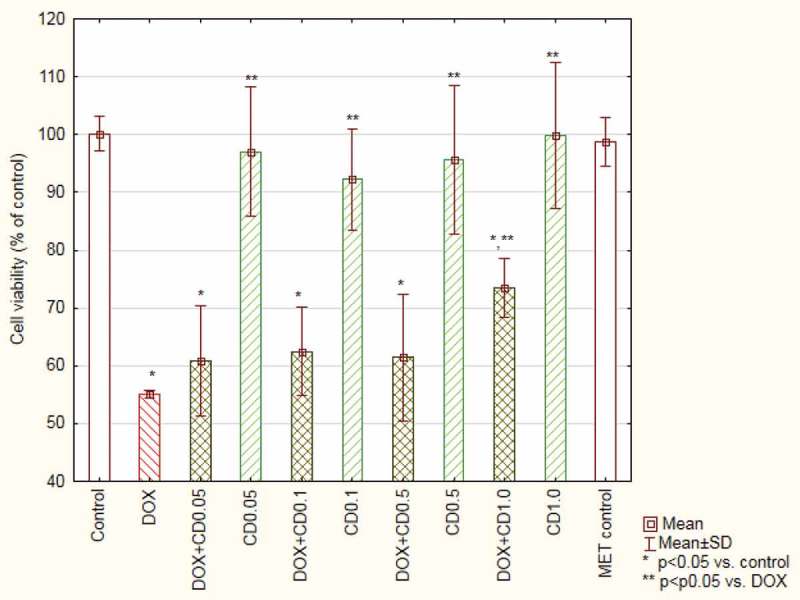
Viability of cardiomyocytes (based on MTT test results) treated with doxorubicin and *Centaurea daghestanica* (Lipsky) Wagenitz extracts. DOX – doxorubicin, CD – *Centaurea daghestanica* (Lipsky) Wagenitz.

The incubation with DOX at a concentration of 5 µM resulted in a decreased cells’ viability to 54.32 ± 0.6% when compared with the control. The simultaneous incubation with the antibiotic and the extracts revealed an increased viability, which is statistically significant in the case of the highest concentration of CD and all tested concentrations of the CB extract. 1 mg/mL of CB resulted in 89.4 ± 1.6% viability of cells incubated with DOX (Figures 3 and 4). Incubation the cells with extracts alone did not change the cells’ viability.

**Figure 5.**
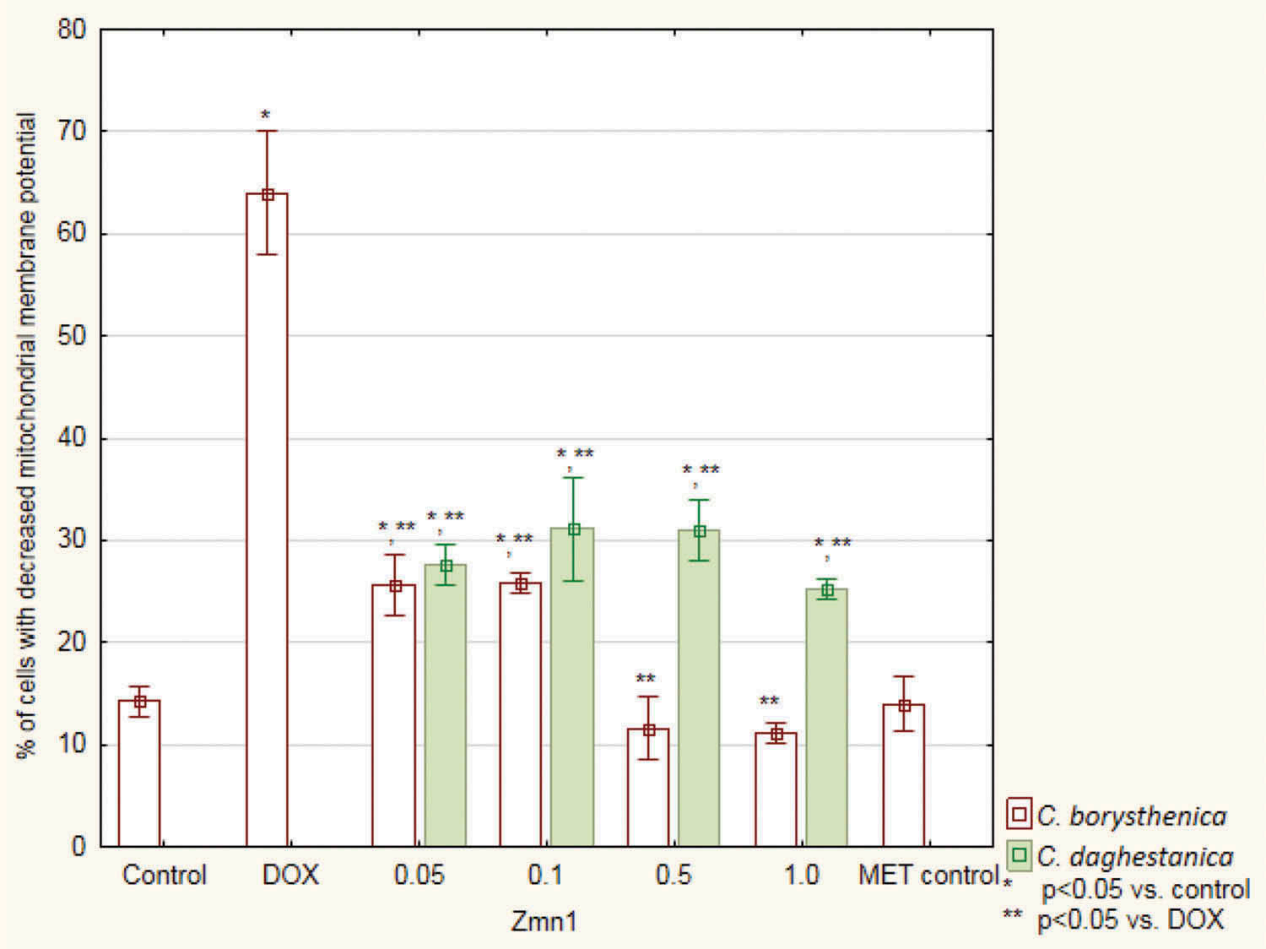
Mitochondrial membrane potential changes based on JC-1 dye transformations. DOX – doxorubicin.

The analysis of changes in the mitochondrial membrane potential of cells treated with DOX showed 65 ± 6% population of cells with reduced potential in comparison to 13 ± 2% in the control. The extracts added to the cultures with DOX caused a significant decrease in the number of cells with reduced potential. The results obtained for two highest concentrations of CB were at the control group’s level (Figure 5).

**Figure 6.**
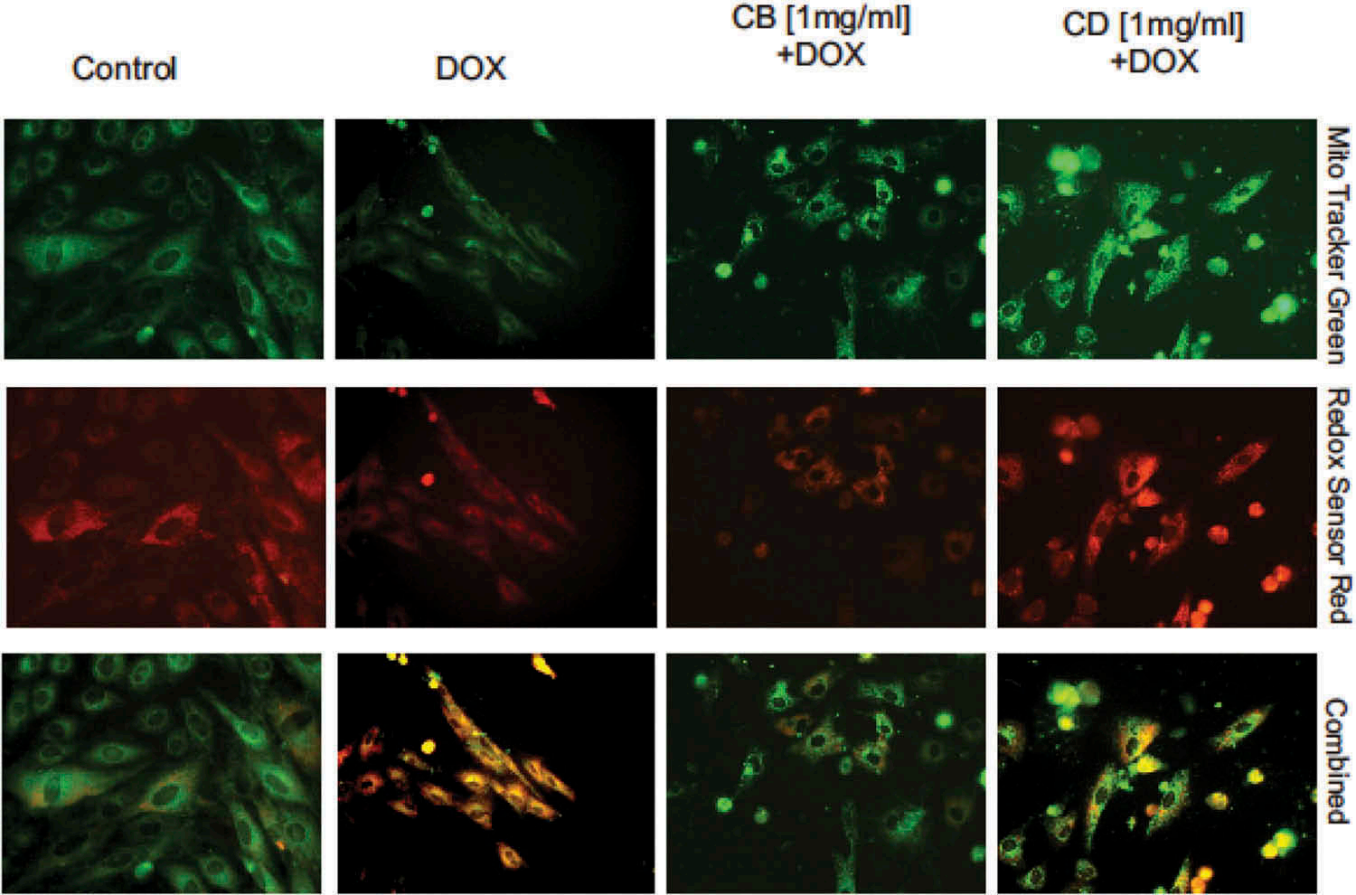
Oxidative stress intensity based on Mito Tracker Green and Redox Sensor Red staining.

In the control and in the control containing CB and CD extracts, a small amount of cells with yellow-orange colour can be noted (Figure 6). In the control of DOX, the intensity of oxidative stress is the greatest. In contrast, the cultures containing DOX and plant extracts showed a lower intensity of oxidative stress, especially in the highest concentration (Table 2).

**Table 2.** The intensity of oxidative stress in cardiomyocytes based on Mito Tracker Green and Redox Sensor Red staining.

	Extract concentration (mg/mL)			
	0.05	0.1	0.5	1.0
DOX+CB	++	++	+	-
DOX+CD	+++	+++	++	+

DOX – doxorubicin, CB – *Centaurea borysthenica* Gruner, CD – *Centaurea daghestanica* (Lipsky) Wagenitz.

### Effect on DOX anti-tumour activity

**Figure 7.**
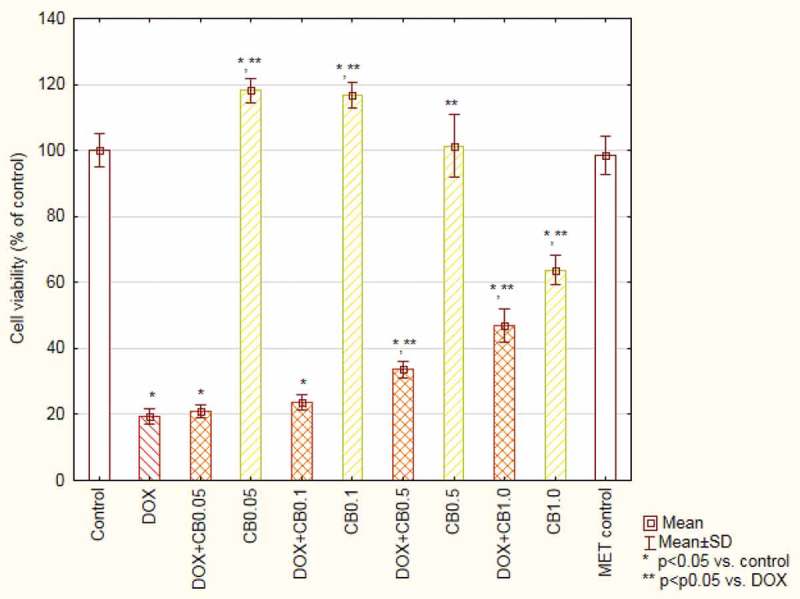
Viability of myeloma cells (based on MTT test results) treated with doxorubicin and *Centaurea borysthenica* Gruner extracts. DOX – doxorubicin, CB – *Centaurea borysthenica* Gruner.

The CB extract at a concentration of 1.0 mg/mL reduced myeloma cell viability to 63.80 ± 4.61%. The extract in lower concentrations has no toxic effect on cancer cells. In the case of concentrations 0.05 and 0.1 mg/mL, the absorbance values were even higher than in control. The viability of cells treated with DOX was 19.40 ± 2.24%. When the cells were treated simultaneously with DOX and CB extracts, significant differences appeared in the case of concentration of 0.5 and 1.0 mg/mL – 33.56 ± 2.62 and 46,96 ± 4.87 % of live cells, respectively (Figure 7).

**Figure 8.**
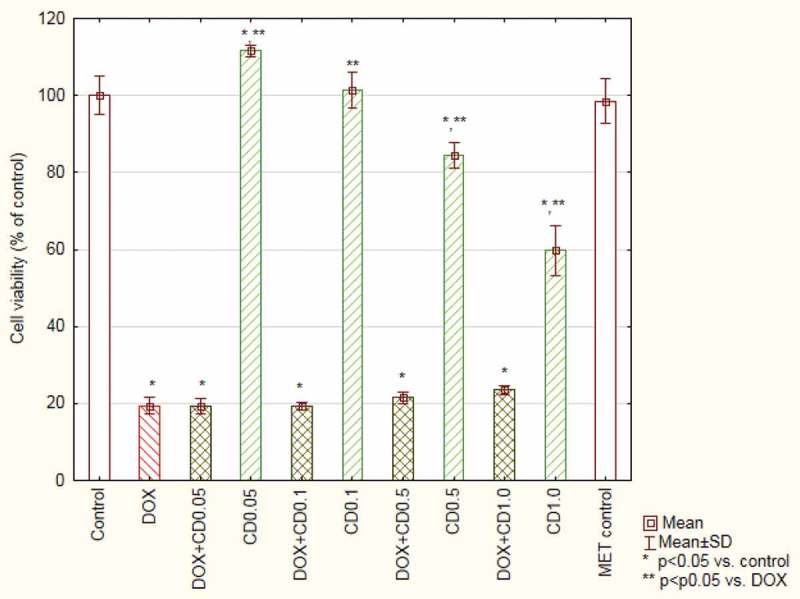
Viability of myeloma cells (based on MTT test results) treated with doxorubicin and *Centaurea daghestanica* (Lipsky) Wagenitz extracts. DOX – doxorubicin, CD – *Centaurea daghestanica* (Lipsky) Wagenitz.

The incubation of myeloma cells with CD extracts resulted in lower viability in the case of 0.5 and 1.0 mg/mL – 84.44 ± 3.29 and 59.7 ± 6.43%. Incubation with DOX and CD did not change the results obtained for DOX only (Figure 8).

## Discussion

DOX is a very efficient anti-tumour drug, but its administration is limited by a dose-dependent, irreversible and progressive cardiomyopathy, which may become evident years after the completion of the therapy [14–16]. The pathomechanism of DOX-related late cardiotoxicity is multifactorial [17,18], but the main role is attributed to oxidative stress connected with redox-cycling of the drug [19,20]. Owing to the great importance of DOX in chemotherapy for the treatment of many types of cancer, researchers have expended great efforts trying to prevent or attenuate the side effects of DOX administration. In this sense, one of the strategies of reducing oxidative stress has been the combination of the drug delivery together with an antioxidant agent [12]. Many antioxidants have been assayed with very different results [21]. Due to their radical-scavenging and iron-chelating properties, flavonoids can be considered as potential protectors against chronic cardiotoxicity caused by DOX. An important property of flavonoids is inhibition of the negative effects of DOX without weakening its anti-tumour activity. Therefore, the preliminary determination of the composition of the extracts obtained predicts the protective effect of cardiomyocytes exposed to DOX.

Extracts from the plant *Centaurea* are an important raw material for medical and pharmaceutical industries. The major compounds located in extracts from *Centaurea* plants are flavonoids, phenolic acids, steroids, triterpenes, and guaianolide. Numerous studies have shown that extracts from the plant *Centaurea* have antioxidant properties [22–25]. Phenolic compounds that belong to the group widely spread in world of plant secondary metabolites have a positive effect on human health. Along with carotenoids, tocopherols, and vitamin C, they are recognized as natural food ingredients with antioxidant properties [26].

In the *Asteraceae* family, flavonoid composition has long been established. The most common flavonoid group includes apigenin, luteolin, and their glucosides, especially 7-glycosides. Polyphenols, particularly flavonoids, have attracted a great deal of research on their broad distribution in plants, their biological (including antioxidant) activities and their health effects. Qualitative and quantitative analysis of the investigated extracts confirmed the presence of apigenin and jacein in two species and apigenin 7-*O*-glucoside (CB) and cynaroside (CD). Moreover, other flavonoid constituents were identified, that revealed biological activity in previous preclinical studies. The composition of phenolic acids and their derivatives is less known [27]. In our study, in the group of phenolic acids, neochlorogenic and protocatechuic acids were identified in two *Centaurea* species. Qualitative and quantitative analysis of the polyphenolic component has been performed in this species for the first time.

In the *Asteraceae* family, the chlorogenic acid is a well-known and well-described compound. The chlorogenic acid is the ester of caffeic and quinic acid. It has a wide, proven spectrum of effects *in vivo* and *in vitro* – antioxidant, anti-inflammatory, anti-HIV, anti-HBV, inhibition of mutagenesis, and carcinogenesis [28–33]. The chlorogenic acid is also a dominant component of the investigated plants’ material, which suggests its significant impact on observed biological effect. Cardiotoxicity *in vitro* analyses showed a protective activity of the methanolic extract of CB in all tested concentrations and the highest concentration of methanolic extracts of CD on rat cardiomyocytes treated with DOX. An important observation is the lack of toxic activity in both extracts in relation to cardiomyocytes. The chlorogenic acid is known for its chemopreventive activities for chemotherapy in cancer patients [34]. A protective effect was also observed in cardiomyocytes treated with DOX in similar *in vitro* model [35]. However, Kan et al. [34] stated that simultaneously it can reduce the cytotoxicity of DOX in cancer cells.

It was revealed in many studies that the mechanism of cardiotoxic action of DOX is connected with the reduction of mitochondrial membrane potential, which leads to apoptosis [5,6]. JC-staining showed a significant increase in the number of cells with reduced mitochondrial potential after DOX treatment. A positive effect of the tested plant extracts on this parameter was observed. Rat model studies have confirmed that stabilization of mitochondrial membrane potential prevents DOX-induced cardiotoxicity [36]. As it was expected, CB and CD methanolic extracts in all concentrations also reduced the severity of oxidative stress induced by DOX.

The second mentioned important feature of cardiotoxicity protective agent is its influence on the anticancer activity of DOX. However, there are reports that the chlorogenic acid which is presented in large quantities in both evaluated extracts, can attenuate DOX cytotoxic activity against cancer cells [34]. For this reason, the influence of the extracts on DOX activity on myeloma cells was evaluated. Although CB is more effective as a cardiomyocyte protective agent, in two higher tested concentrations it weakened the drug activity. The CD extract did not change DOX efficacy in the evaluated experiment, even in the highest concentration.

Interestingly, it was observed that both tested extracts are cytotoxic for myeloma cells and in the highest concentration they reduce cells’ viability to about 60%. An anti-proliferative activity has been found in other species of *Centaurea* in recent years [37–40]. Different types of secondary metabolites (flavonoids, sesquiterpenes) were found to be responsible for the antitumour effects of the extracts. Interestingly, the flavone that was found in both species – apigenin – is known for its anti-tumour activity, as well as synergistic effect with conventional chemotherapeutic agents [41,42]. For this reason, it seems reasonable to examine more accurately the obtained extracts of two *Centaurea* species in this regard.

In conclusion, the detected antioxidant activity of extracts of *C. borysthenica* Gruner and *C. daghestanica* (Lipsky) Wagenitz can be used in the prevention of DOX-induced cardiotoxicity. However, the method of application and the optimal dose need further investigation.
